# Aligning implementation science with improvement practice: a call to action

**DOI:** 10.1186/s43058-021-00201-1

**Published:** 2021-09-08

**Authors:** Jennifer Leeman, Catherine Rohweder, Matthew Lee, Alison Brenner, Andrea Dwyer, Linda K. Ko, Meghan C. O’Leary, Grace Ryan, Thuy Vu, Shoba Ramanadhan

**Affiliations:** 1 School of Nursing, The University of North Carolina at Chapel Hill, CB #7460, Chapel Hill, NC 27599 USA; 2 Center for Health Promotion & Disease Prevention, The University of North Carolina at Chapel Hill, CB #7424, Carrboro, NC 27510 USA; 3 Department of Population Health, NYU Grossman School of Medicine, 180 Madison Ave, 8th Floor, New York, NY 10016 USA; 4 Department of General Medicine & Clinical Epidemiology, UNC School of Medicine, The University of North Carolina at Chapel Hill, CB #7293, Carrboro, NC 27510 USA; 5 University of Colorado Cancer Center, 13001 East 17th Avenue, Aurora, CO 80045 USA; 6 Department of Health Systems and Population Health, School of Public Health, University of Washington, Seattle, USA; 7 Department of Cancer Prevention, Fred Hutchinson Cancer Research Center, Hans Rosling Center for Public Health, 3980 15th Avenue NE, 4th Floor, Seattle, WA 98195 USA; 8 Department of Health Policy and Management, Gillings School of Global Public Health, The University of North Carolina at Chapel Hill, CB #7400, Chapel Hill, NC 27599 USA; 9 The University of Iowa, 145 N. Riverside Drive, N475 CPHB, Iowa City, IA 52242 USA; 10 Department of Social and Behavioral Sciences, Harvard T.H. Chan School of Public Health, 677 Huntington Ave, Boston, MA 02115 USA

**Keywords:** Implementation science, Improvement practice, Quality improvement, Cancer prevention and control, Case studies

## Abstract

**Background:**

In several recent articles, authors have called for aligning the fields of implementation and improvement science. In this paper, we call for implementation science to also align with improvement practice**.** Multiple implementation scholars have highlighted the importance of designing implementation strategies to fit the existing culture, infrastructure, and practice of a healthcare system. Worldwide, healthcare systems are adopting improvement models as their primary approach to improving healthcare delivery and outcomes. The prevalence of improvement models raises the question of how implementation scientists might best align their efforts with healthcare systems’ existing improvement infrastructure and practice.

**Main body:**

We describe three challenges and five benefits to aligning implementation science and improvement practice. Challenges include (1) use of different models, terminology, and methods, (2) a focus on generalizable versus local knowledge, and (3) limited evidence in support of the effectiveness of improvement tools and methods. We contend that implementation science needs to move beyond these challenges and work toward greater alignment with improvement practice. Aligning with improvement practice would benefit implementation science by (1) strengthening research/practice partnerships, (2) fostering local ownership of implementation, (3) generating practice-based evidence, (4) developing context-specific implementation strategies, and (5) building practice-level capacity to implement interventions and improve care. Each of these potential benefits is illustrated in a case study from the Centers for Disease Control and Prevention’s Cancer Prevention and Control Research Network.

**Conclusion:**

To effectively integrate evidence-based interventions into routine practice, implementation scientists need to align their efforts with the improvement culture and practice that is driving change within healthcare systems worldwide. This paper provides concrete examples of how researchers have aligned implementation science with improvement practice across five implementation projects.

**Supplementary Information:**

The online version contains supplementary material available at 10.1186/s43058-021-00201-1.

Contributions to the literature

- Tension continues to exist between the fields of implementation science and improvement practice. This paper presents both the challenges and benefits to aligning the two fields.
- This paper argues that greater alignment between implementation science and improvement practice has the potential to strengthen the implementation of evidence-based interventions.
- Five case studies provide concrete examples of how Cancer Prevention and Control Research Network members are achieving the benefits of aligning implementation science with improvement practice.

## Background

In several recent articles, authors have called for aligning the fields of implementation and improvement science with the goal of strengthening both fields’ potential to improve care [1–3]. These authors contend that closer alignment would promote “cross-fertilization,” with each field learning from and building on the other’s expertise, methods, and frameworks [2]. While we recognize the value of closer alignment between implementation science and improvement science, we contend that implementation science may gain as much or more from greater alignment with improvement practice.

Implementation science and improvement science both aim to produce generalizable knowledge to guide the improvement of healthcare services and health outcomes but differ in their approach. Implementation science focuses on increasing the uptake of evidence-based interventions (EBIs) whereas improvement science focuses on iteratively measuring and improving care processes and systems (2). Improvement science has its foundation in the field of improvement practice, which also focuses on improving care processes and systems (1). Improvement practice is distinct from improvement science in that its goal is to produce the local (rather than generalizable) knowledge needed to improve care in a specific setting [1]. In this paper, we acknowledge other scholars’ observations on the value of aligning implementation science and improvement science: for example, the opportunity to share their respective expertise in assessing contextual factors and engaging practice-level stakeholders in the co-creation of solutions [2]. However, we move beyond this prior work by highlighting the need for greater alignment between implementation science and improvement practice, with improvement practice defined as practice-initiated efforts to design and redesign work processes and systems with the goal of improving local outcomes [4].

Across the USA and worldwide, healthcare systems are adopting Lean Six Sigma, quality improvement, and other improvement models as their primary approach to improving practice [5–10]. Table 1 provides a list of widely used improvement models [11].

**Table 1.** Sample improvement models^a^

Quality improvement model	
Continuous Quality Improvement (CQI)	
Donabedian Model	
European Foundation for Quality Management (EFQM) model	
ISO 9000-9004 (International Organization for Standardization)	
Joint Commission on Accreditation of Healthcare Organizations (JCAHO) Model	
Kooperation für Transparenz und Qualität im Krankenhaus (KTQ)	
Lean Management	
Malcolm Baldrige Quality Award (MBQA)	
Quality Improvement (QI)	
Six Sigma	
Strategic Collaborative Quality Management (SCQM)	
Total Quality Management (TQM)	

^a^ Models were referenced at least 3 times according to [9, 11]

*EBI* evidence-based intervention, *PDSA* Plan-Do-Study-Act, *HPV* human papillomavirus, *FIT* fecal immunochemical test

The widespread adoption of improvement models is driven in part by requirements that health systems develop improvement programs to qualify for health insurance reimbursement and other sources of funding [12–15]. Concurrently, a host of organizations have emerged to provide training and online resources to build improvement capacity [16–20]. As a result, many healthcare systems are investing in improvement infrastructure, hiring personnel with expertise in improvement models, and training providers and staff to use tools such as root cause analysis, process flow diagramming, run charts, and Plan-Do-Study-Act (PDSA) cycles [21–25].

A central tenet of implementation science is that implementation strategies will be most successful when they align with healthcare systems’ existing culture, infrastructure, and practices [26]. Consistent with this tenant, healthcare systems’ widespread adoption of improvement models raises the question of whether and how implementation scientists might best align their efforts with improvement practice. In this paper, we describe three challenges and five benefits of aligning implementation science with improvement practice. We then illustrate each of the potential benefits of alignment in five case studies from the Centers for Disease Control and Prevention (CDC)-funded Cancer Prevention and Control Research Network (CPCRN).

## Main text

### The challenges of aligning implementation science with improvement practice

As detailed below, three primary challenges have impeded the alignment of implementation science with improvement practice.

#### Challenge 1: Implementation science and improvement practice use different models, terminology, and approaches

Implementation science and improvement practice both promote specific approaches, referred to as “strategies” in implementation science and “interventions” or “tools” in improvement practice [1, 2]. While the approaches have similar objectives, the two fields use different terminology and apply different techniques. For example, both implementation science and improvement practice promote approaches for identifying contextual factors that contribute to a problem and/or inform a practice change. Both fields also promote strategies, interventions, and tools for use in developing and testing solutions to problems. The implementation science literature describes a multitude of strategies, with one widely cited taxonomy listing 73 distinct strategies [27]. In contrast, each improvement model offers its own distinct and relatively small list of interventions and tools, such as Fishbone diagrams, the Five Why’s, and process flow diagrams among others [28]. Although many of these interventions and tools are similar across models, they may vary in terminology, format, methods, and frameworks. These variations not only create barriers to communication among implementation scientists and improvement practice professionals, but also create confusion among the frontline providers and staff who are central to the hands-on work of implementing EBIs.

#### Challenge 2: Improvement practice produces locally specific, rather than generalizable, knowledge

As noted above, implementation science and improvement practice have different objectives. Implementation science aims to provide generalizable knowledge to improve healthcare services and outcomes. In contrast, improvement practice seeks to generate local knowledge to improve healthcare services and outcomes in a specific setting [1]. The emphasis given to the difference between science and practice contributes to the tendency to view improvement practice as outside of implementation science’s scope. For example, Mitchell and Chambers (2017) depict improvement science, implementation science, and other fields as forming an inter-related grouping of disciplines, and place quality improvement (a widely used improvement model) in a small oval completely outside that grouping [29].

#### Challenge 3: The evidence base for the effectiveness of improvement practice is limited

Systematic reviews of the literature have identified wide variation in how healthcare systems are using improvement models and tools [30–32]. For example, Taylor and colleagues [30] reviewed studies that used Plan-Do-Study-Act (PDSA cycles), a widely used improvement tool that involves multiple, small, rapid tests of a local improvement. They found that 81% (59/73) of studies failed to report the conduct of iterative cycles of change, and only two of the studies reviewed demonstrated healthcare system compliance with all key principles of the PDSA cycle method [30]. Reed and Card (2016) determined that teams rarely completed the full PDSA cycle, putting most of their effort into “Doing” and far less into “Studying” and “Acting” [31]. In a systematic review of the literature, Hill et al. (2020) found mixed evidence for the effect of quality improvement on health services and little evidence for effects on health outcomes [32]. The limited evidence in support of effectiveness may lead implementation scientists to dismiss improvement practice as an approach that does not work. However, this is not the conclusion of Hill et al. who point to the poor quality of evaluation methods, complexity of the changes being evaluated, and the need for more well-designed studies to demonstrate quality improvement’s effectiveness [32].

### The benefits of aligning implementation science with improvement practice

In this paper, we advocate for the benefits of implementation science and improvement practice aligning with and building on each other’s strengths. We do not argue for the value of generalizing the findings from improvement projects or for the effectiveness of improvement practice at improving health services and outcomes. Rather, we highlight five potential benefits of aligning with improvement practice as a means of strengthening research/practice partnerships, fostering local ownership of EBI implementation, generating in-depth knowledge of the practice context, and developing context-specific implementation strategies.

#### Benefit 1: Strengthen research/practice partnerships by supporting healthcare systems’ improvement projects

Implementation science typically requires that researchers engage with healthcare providers, staff, and multiple levels of system leadership in the healthcare systems where they plan to implement EBIs. Engaging these stakeholders is challenging due to competing demands for their time and attention. Furthermore, these stakeholders may resist the input of researchers outside their organizations, as reflected in a recent article where authors refer to implementation science as tending toward “scientific imperialism” and an “overall approach that disrespects and undervalues primary care as a co-producer of knowledge” [33]. This observation not only applies to primary care but also to other healthcare systems and providers. As noted above, many healthcare systems are adopting improvement models as their primary method for improving care processes. Recognizing and respecting this reality may be key to implementation scientists gaining entrée into healthcare systems and engaging stakeholders in the adoption, adaptation, and implementation of new EBIs [34]. In addition to supporting implementation research, engaged research/practice partnerships can strengthen healthcare systems’ improvement projects by contributing expertise in potential solutions to identified problems and methods for evaluating impact.

#### Benefit 2: Foster local ownership of EBI implementation and sustainment

In improvement teams, providers and staff work together to plan and test improvements in their unique healthcare setting, thereby promoting local ownership of the change in practice [35]. Partnerships between improvement teams and implementation scientists can yield opportunities to co-create EBIs as well as implementation strategies. Decades of research have shown that individuals are more likely to adopt and implement interventions when they view themselves as the owners of the interventions and are able to shape them to fit local needs and preferences [36]. Local ownership also is essential to sustaining an intervention over time. Once engaged, an improvement team has the potential to monitor implementation and respond to changes such as new regulations, new funding streams, or changes in staffing or in the population served [37].

#### Benefit 3: Employ improvement tools to generate practice-based evidence

Eliciting local stakeholders’ knowledge of their local context is key to ensuring that an EBI and its implementation align with a practice setting’s needs, assets, and priorities. Recent years have seen a growing emphasis on the importance of this type of “practice-based” evidence, which Leeman and Sandelowski defined as “evidence concerning the contexts, experiences, and practices of healthcare providers working in real-world practice settings” [38]. Improvement models (e.g., Lean Six Sigma, the Improvement Model) typically consist of a systematic, multistep approach that involves the use of specific tools to describe current processes (e.g., process maps) and identify the root causes of problems with or gaps in those processes (e.g., fishbone diagrams). These tools are designed to capture team members’ local knowledge of the factors that influence care delivery and outcomes within a specific setting and population. In this way, practical considerations drive the selection, testing, and refinement of candidate solutions for a given evidence-practice gap. By partnering with improvement teams on the use of process maps and other improvement tools, implementation scientists can elicit the practice-based evidence needed to select, adapt, and refine EBIs and implementation strategies [39]. This careful attention to local knowledge may be particularly important to efforts to promote health equity, as it is key to identifying factors that contribute to health disparities [40].

#### Benefit 4: Conduct Plan-Do-Study-Act (PDSA) cycles to develop and refine implementation strategies

Another primary component of many improvement models is the PDSA cycle. In conducting PDSA cycles, improvement teams design changes that they then rapidly test in multiple, small experiments with the goal of developing “fit-for-purpose solutions” to improve care [30]. In contrast, many of the current approaches to developing implementation strategies require substantial, upfront investments of time and resources (e.g., Implementation Mapping) [41, 42]. Furthermore, they often yield a multicomponent implementation strategy that is then “frozen” and distributed for replication across multiple settings [37]. While settings may be encouraged to adapt multicomponent implementation strategies, little guidance is provided on how to identify and address contextual factors that may influence implementation in new settings [43]. PDSA cycles offer an approach that implementation scientists might apply to engage local improvement teams in rapid, iterative testing of implementation strategies. Multiple examples exist of implementation scientists using PDSA cycles to develop, test, and refine implementation strategies, such as in the case of “implementation laboratories” [44] and multi-organization “learning evaluation” projects [45].

#### Benefit 5: Build healthcare systems’ overall capacity to implement EBIs and improve care

Strengthening healthcare systems’ use of improvement models and tools is important for both the improvement teams and implementation scientists to achieve their maximum potential. The literature includes examples of implementation scientists partnering with healthcare systems to bolster application of their improvement models and tools, usually in support of a specific EBI [46]. Efforts to strengthen a system’s use of improvement models and tools include the provision of training, tools, and other support to build improvement capacity as well as evaluations of improvement processes and outcomes. Of note, efforts to build improvement capacity may incorporate implementation science frameworks, strategies, and outcomes. For example, evaluations of improvement projects may draw on implementation frameworks and outcomes. In addition, programs intended to strengthen the use of improvement models and tools may include menus of implementation strategies to broaden the solutions available to overcome barriers to improvement.

### Cancer Prevention and Control Research Network (CPCRN) case studies of implementation science/improvement practice alignment

**Fig. 1:**
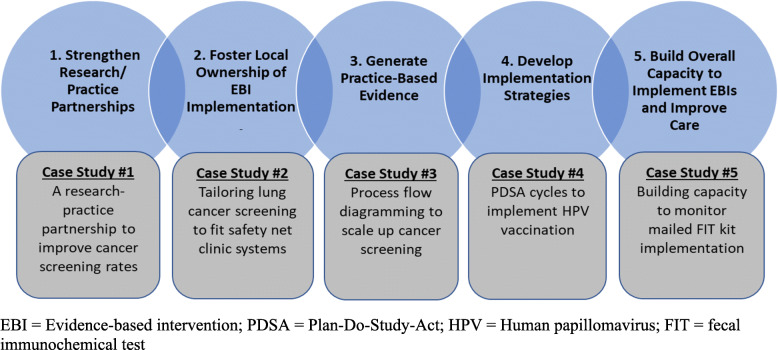
Five case studies illustrating the five benefits of aligning implementation science and improvement practice within cancer prevention and control

As summarized in Fig. 1, we describe five case studies that illustrate how CPCRN researchers are achieving the benefits of aligning implementation science and improvement practice in their cancer prevention and control projects. Each of the case studies was selected to highlight one of the five benefits; however, in reality each case exemplifies multiple benefits.

The CPCRN is a national network funded by the Centers for Disease Control and Prevention (CDC) and National Cancer Institute (NCI). Since 2002, CPCRN centers have conducted practice-engaged research to accelerate the adoption and implementation of cancer prevention and control interventions [47, 48]. In this current funding cycle (2019–2024), the CPCRN created a cross-center workgroup to study the alignment of implementation science and improvement practice. Workgroup members from the eight CPCRN member sites completed an initial survey of implementation research projects that incorporated improvement models and tools. A total of 17 projects were identified and described. Below, we highlight five case studies, each of which was selected to illustrate one of the benefits of aligning implementation science and improvement practice.

#### Case Study 1: Utilizing a research/practice partnership to improve colorectal cancer screening rates (Benefit: Strengthen research/practice partnerships)

Researchers at the University of North Carolina (UNC) Chapel Hill are partnering with a large, academic primary care practice that serves 13,000 adult patients and provides over 39,000 visits per year. The practice has extensive experience with quality improvement (QI) and participates in a system-wide improvement collaborative.

**Fig. 2:**
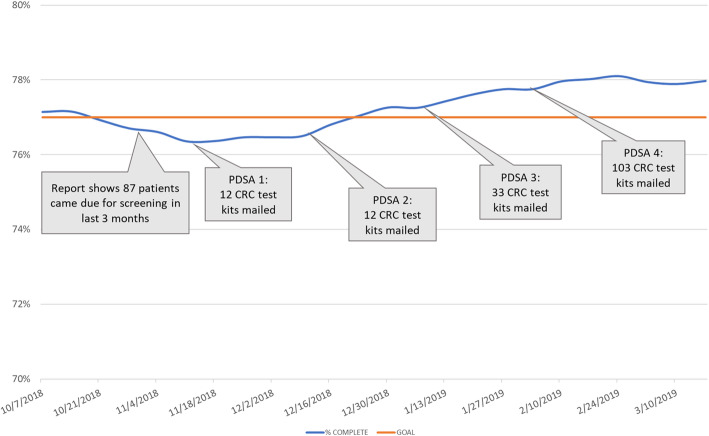
Run chart of CRC test kit completion rates

Recently, UNC researchers partnered on a clinic-initiated project to address a sudden drop in colorectal cancer (CRC) screening rates. In October 2018, the practice’s QI committee noted a steep decrease in CRC screening rates while reviewing standard clinical quality measures. A subgroup of the QI committee was formed to study the problem and implement potential solutions. The team conducted a root-cause analysis and found that prior interventions, in which CRC screening home test kits were distributed during annual clinic visits, increased CRC screening rates. However, the interventions were not sustained over time, resulting in a drop in screening rates. To identify a more sustainable solution, the team engaged UNC researchers with expertise in mailed CRC test kit outreach, a CRC screening intervention that does not require a clinic visit and has limited impact on clinic workflows. Researchers partnered with the team on multiple PDSA cycles to develop a protocol for mailing CRC test kits, targeting patients who had previously completed a screening kit but were now overdue (Fig. 2). From November 2018 to April 2019, eligible patients received (a) a mailed packet containing an introductory letter and a screening kit, and (b) a reminder message through the patient portal. A total of 160 screening kits were mailed across four waves. In the first three waves, adjustments were made between mailings to improve the materials (e.g., wave 2 included an edited introductory letter, wave 3 included a Spanish language letter). As illustrated in Fig. 2, the project achieved its goal of increasing CRC screening rates to above 77%. This case study illustrates the importance of researcher support for a practice-initiated project in developing strong research/practice partnerships. Through their decade-long partnership with this clinic, CPCRN researchers have engaged clinic stakeholders in multiple researcher-initiated projects [49, 50].

#### Case Study 2: Tailoring lung cancer screening to fit clinic systems within the Colorado Cancer Screening Program for Patient Navigation (Benefit: Foster local ownership of EBI implementation)

Researchers and public health practitioners at the University of Colorado Cancer Center and the Colorado School of Public Health have developed and implemented the Colorado Cancer Screening Program (CCSP). With funding from state-level tobacco tax revenue, the CCSP has built extensive statewide partnerships, including partnerships with nearly 150 clinic systems and other organizations. The cornerstone of CCSP over the last decade has been the use of patient navigation to deliver multi-level EBIs to increase CRC screening. Patient navigation is an evidence-based intervention that employs patient navigators to guide patients through the health care system and reduce barriers to screening and treatment [51]. In 2018, CCSP partnered with National Jewish Health to develop training, materials, and tools to expand patient navigation to lung cancer screening. In 2019, CCSP invited members of their statewide network of clinic systems to pilot the materials and tools. Guided by the PRISM framework [52], the CCSP team conducted an assessment to determine clinic readiness and selected two community health centers with the organizational capacity (i.e., commitment to cancer screening and staffing resources and time) and environmental supports (i.e., access to hospital or radiology practices to provide low-dose computed tomography [CT] screening services) needed to pilot test the lung cancer screening materials and tools. One of the tools was a process flow template outlining the processes that clinics and patient navigators would use to identify patients eligible for screening, engage providers and patients in shared decision making, refer patients to CT lung cancer centers/hospitals, and track processes and outcomes [53, 54].

**Fig. 3:**
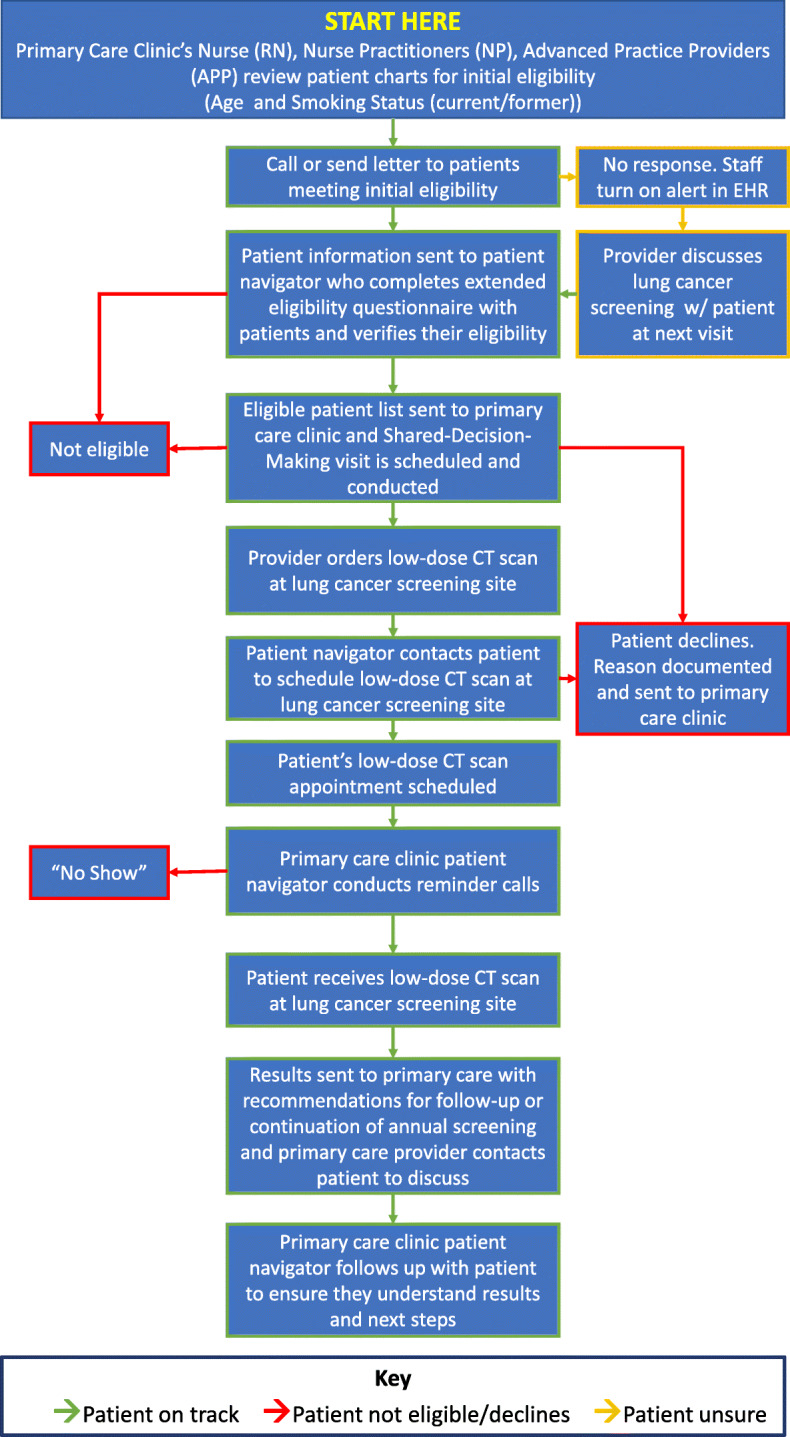
Process flow diagram for lung cancer screening

CCSP provided technical assistance to aid clinics in adapting the process flow template and creating a process flow diagram that was tailored to each clinic’s existing staffing, workflows, and referral arrangement with CT lung cancer centers/hospitals. For example, the more urban of the two pilot clinics shares an electronic health record (EHR) with its CT provider. This shared system allows their primary care team to have a streamlined process flow diagram for referring patients and tracking processes and outcomes (Fig. 3). In contrast, the rural clinic does not have a formal connection with its CT provider nor do they share the same EHR. As a result, a more complex process flow diagram was needed to outline the processes needed to refer patients and track outcomes (see Additional File 1: Lung Cancer Screening Workflow). The approach to customizing workflows, while maintaining fidelity to the guidelines, allows the decision makers within each system to embrace lung cancer screening and implement it within existing capacity and resources while also planning for longer-term sustainability of their efforts.

#### Case Study 3: Diagramming process flow to scale up colorectal cancer screening (Benefit: Generate practice-based evidence)

UNC researchers are incorporating process flow diagramming throughout the design, implementation, and evaluation of their National Institutes of Health (NIH)-funded Scaling Colorectal Cancer Screening through Outreach, Referral, and Engagement (SCORE) study. In collaboration with two community health centers, SCORE is testing the effectiveness of an intervention that combines mailed CRC screening home test kits, patient reminders, and navigation to follow-up colonoscopy for patients with a positive test result. Process flow diagrams are being used to highlight key differences in the process steps and to delineate roles and resources needed based on feedback from clinic staff about their local context and standards of care. The research team iteratively developed the process maps through multiple stakeholder-engaged sessions. The maps reflect input from program directors and staff at the community health centers where SCORE is being implemented. Each diagram documents all steps in the process and identifies which individuals perform each step. The team created a separate diagram for each of the core components of the SCORE intervention, which include developing a registry of eligible patients, conducting a mailed screening program, and navigating patients with a positive screening test to follow-up colonoscopy.

Early iterations of the process flow diagrams were used to plan implementation of the intervention. Visualization of the planned steps helped identify outstanding questions about both the intervention and implementation, as well as prioritize decision-making. These decisions included (a) determining the total number of patient contacts (e.g., mail and phone reminders); (b) optimizing the order of intervention activities and the time intervals between activities; and (c) identifying the appropriate individual or organization to perform specific steps. The process flow diagrams were then used to develop a comprehensive set of implementation outcomes to be measured. For example, fidelity and cost measures were mapped onto each process step, noting which tool(s) would be used for measurement, the frequency of measurement, and where these data would be tracked. The ongoing assessment of these measures during implementation will provide insight into the time and resources required for implementation, potential challenges to implementing the intervention as intended, and opportunities to create efficiencies. The evidence gathered from the SCORE process flow diagrams will be used to inform the adaptation and sustainment of the intervention in community health centers across the state.

#### Case Study 4: Using Plan-Do-Study-Act cycles to implement human papillomavirus vaccination programs (Benefit: Develop implementation strategies)

Researchers at the NYU Grossman School of Medicine are partnering with six community health centers (three pediatric clinics and three school-based clinics) in Southwest Brooklyn to evaluate and support the implementation of their HPV vaccination programs for low-income adolescent populations. As a first step, semi-structured key informant interviews are being conducted with multi-level stakeholders, including medical directors and other organizational leaders, nurse practitioners, frontline staff, and administrators across all six clinics. Guided by the RE-AIM framework [55], these interviews serve the dual purposes of establishing and maintaining relationships with organizational leaders and staff across the community health centers and soliciting multi-level perspectives on organizational processes and outcomes regarding the adoption, implementation, effectiveness, and sustainability of their HPV vaccination efforts. Analyses of the interview data will include understanding and documenting: (a) improvements efforts and infrastructure that are currently in place at each clinic; (b) implementation strategies that are being used to support reach, retention, and equity; and (c) ideas for how to improve the programs moving forward both in general and in response to the COVID-19 pandemic.

Informed by findings from these key informant interviews, the NYU team will convene a virtual learning collaborative and provide technical assistance to support each community health center as they conduct iterative PDSA cycles to refine existing implementation strategies and develop new ones as needed to address barriers to implementation. In addition to PDSA cycles, clinics will learn to use additional improvement tools that guide root cause and strength/weakness/opportunity/threat (SWOT) analyses (e.g., fishbone diagrams) for use in identifying and addressing potential threats as community health centers seek to build capacity for and sustain high-quality HPV vaccination programs in the future.

#### Case Study 5: Building capacity to monitor the implementation of mailed CRC screening home test kits (Benefit: Build overall capacity)

Researchers at the University of Washington (UW) provide implementation and evaluation technical assistance to community health centers across Washington State that participate in the CDC’s Colorectal Cancer Control Program. In this role, UW researchers partnered with three community health centers to evaluate a mailed CRC screening home test kit intervention to increase CRC screening. The three community health centers operate 45 medical clinics that mail out more than 2000 CRC test kits annually. UW researchers met individually with community health centers to learn about their CRC screening workflows and identify data capture points for monitoring and improvement. During these visits, community health center partners reported that their current quality monitoring systems lacked the capacity and flexibility to comprehensively monitor and evaluate implementation of their mailed screening kit programs.

Researchers developed a simple Microsoft Excel tracking tool for partners to use; tool contents were tailored based on one-on-one consultations with each community health center to align with their needs and resources. The tool included patient-level implementation variables to track such as demographic data, key dates (e.g., initial mailing date, reminder date(s), date screening kit was returned), test result, referral to colonoscopy (if needed), and colonoscopy outcome. Researchers pre-programmed reports in the tool so that community health centers could generate reports to monitor implementation in real time. Feedback from community health center staff on the tool and its adaptations has been predominantly positive. One community health center was able to work with their EHR vendor to add some of the suggested implementation measures directly into their EHR. A second community health center made enhancements to the tool when it became clear that their system had significant gaps in closing the colonoscopy referral loop and recording follow-up data. The third community health center reported that the automated reports enhanced their ability to provide real-time reporting to leadership, whereas previous reports were less timely due to data lags caused by processing internal data requests. In turn, community health center partners have supported UW’s research efforts over the past 5 years by providing letters of support for research applications and participating in emergent research opportunities.

## Conclusion

The number of EBIs available to prevent and control cancer continues to increase [56, 57], and healthcare systems need support to fully implement and sustain these life-saving interventions and programs over time. In this paper, we contend that the alignment of implementation science with improvement practice has the potential to improve EBI implementation and sustainment. We describe five benefits of this alignment and present five case studies to illustrate how CPCRN researchers are leveraging improvement practice to implement CRC screening, lung cancer screening, and HPV vaccination EBIs in diverse healthcare settings. While we aligned each case study with a single benefit for clarity, the projects actually achieved multiple, overlapping benefits. Most notably, all cases resulted in stronger research/practice partnerships and increased capacity to implement EBIs and improve care.

Investments are needed to build the capacity within both implementation science and improvement practice. Ideally, implementation scientists would include orientation to improvement practice in the multiple trainings being offered to build capacity in the field of implementation science [58] and would encourage attendees to participate in one or more of the many trainings on improvement practice [16]. To build practice-level capacity, implementation scientists might “embed” themselves within healthcare systems’ improvement efforts [59, 60]. As illustrated in this paper’s case studies, implementation scientists, often in partnership with other intermediary organizations (e.g., American Cancer Society, National Jewish Health), provide learning collaboratives, training, and technical assistance to support clinics’ use of EBIs and quality improvement models and tools. The case studies illustrate how researchers began by assessing partners’ capacity (or readiness) to use improvement models and tools and then tailored their training and other strategies accordingly. By doing so, they built practice partners’ capacity to use improvement tools and models to implement and sustain EBIs over time. In our examples, the improvement tools that CPCRN researchers supported included run charts, process flow diagrams, root cause analysis, quality monitoring systems, and PDSA cycles. Most importantly, the case studies illustrate how positive outcomes can be achieved when both implementation scientists and improvement practitioners center their efforts on a common improvement goal, and draw on the full breadth of their respective expertise to achieve that goal.

## Supplementary Information

**Additional file1:.**

## Data Availability

Not applicable to this article as no datasets were generated or analyzed during the current study.

## Abbreviations

ACS: American Cancer Society
CCSP: Colorado Cancer Screening Program
CDC: Centers for Disease Control and Prevention
CPCRN: Cancer Prevention and Control Research Network
CQI: Continuous quality improvement
CRC: Colorectal cancer
CT: Computed tomography
EBI: Evidence-based intervention
EFQM: European Foundation for Quality Management
EHR: Electronic health record
HPV: Human papillomavirus
ISO: International Organization for Standardization
JCAHO: Joint Commission on Accreditation of Healthcare Organizations
KTQ: Kooperation für Transparenz und Qualität im Krankenhaus
MBQA: Malcolm Baldrige Quality Award
NCI: National Cancer Institute
NIH: National Institutes of Health (PDSA) Plan-Do-Study-Act
QI: Quality improvement
SCORE: Scaling Colorectal Cancer Screening through Outreach, Referral, and Engagement
SCQM: Strategic Collaborative Quality Management
SWOT: Strength/Weakness/Opportunity/Threat
TQM: Total Quality Management
UI: University of Iowa
UNC: The University of North Carolina
UW: University of Washington
