# Supplementary figures and images for: Aligning implementation science with improvement practice: a call to action
Source: Implement Sci Commun. 2021 Sep 8;2:99. doi: 10.1186/s43058-021-00201-1 (PMC8424169; doi:10.1186/s43058-021-00201-1)

**
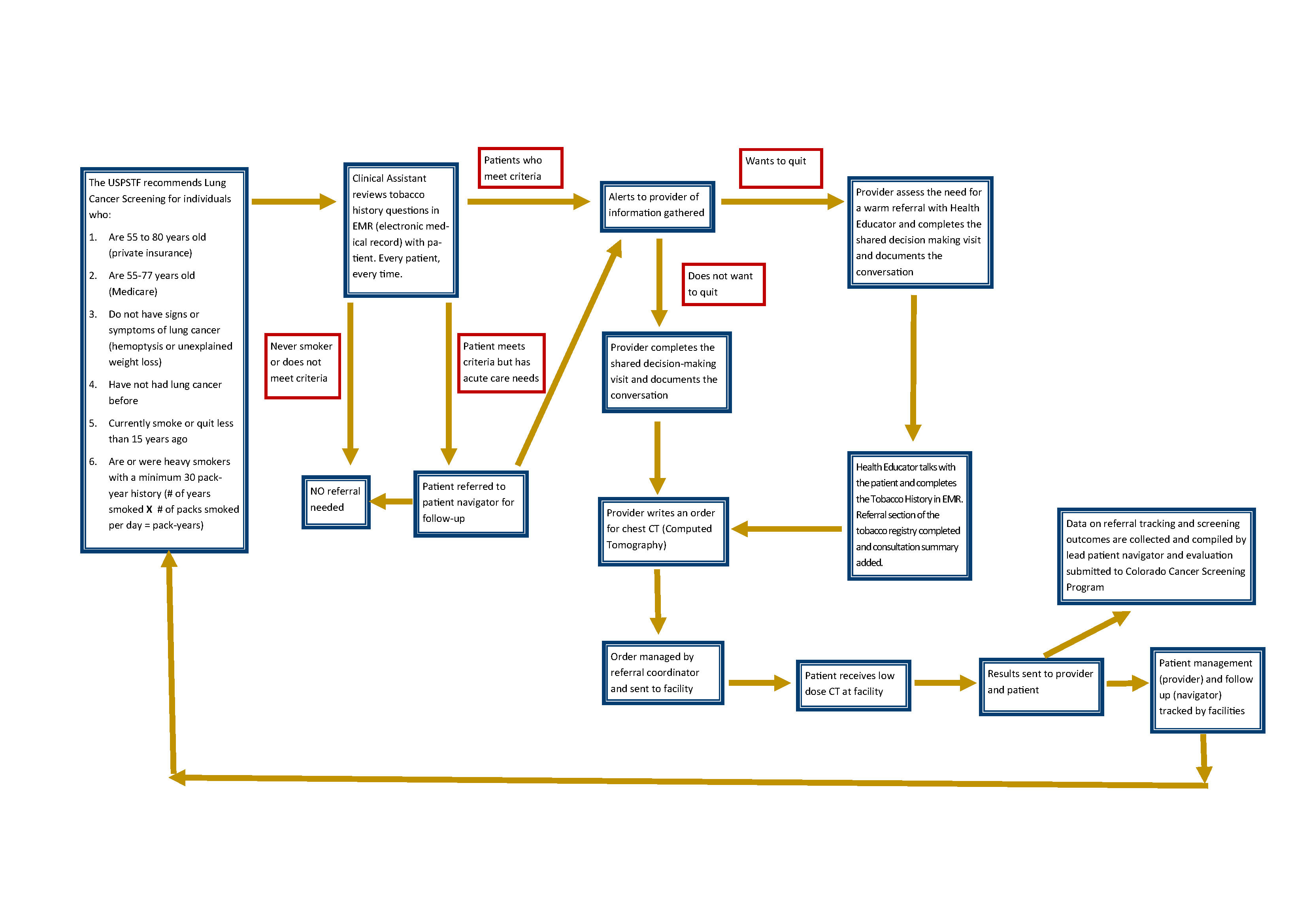
Additional File 1: Lung Cancer Screening Workflow**

Supplement: Supplementary file 1 — Additional file1:. [file 43058_2021_201_MOESM1_ESM.docx]
